# Serotonin: an overlooked regulator of endocytosis and endosomal sorting?

**DOI:** 10.1242/bio.059057

**Published:** 2022-01-25

**Authors:** Gregory Redpath, Nikita Deo

**Affiliations:** 1 EMBL Australia Node in Single Molecule Science, School of Medical Sciences and the ARC Centre of Excellence in Advanced Molecular Imaging, University of New South Wales, Sydney 2052, Australia; 2 Department of Biochemistry, University of Otago, Dunedin 9016, New Zealand

**Keywords:** Serotonin, Serotonin receptor, Serotonin transporter, Serotonylation, Endocytosis, Endosomal sorting

## Abstract

Serotonin is a neurotransmitter and a hormone that is typically associated with regulating our mood. However, the serotonin transporter and receptors are expressed throughout the body, highlighting the much broader, systemic role of serotonin in regulating human physiology. A substantial body of data strongly implicates serotonin as a fundamental regulator of endocytosis and endocytic sorting. Serotonin has the potential to enhance endocytosis through three distinct mechanisms – serotonin signalling, serotonylation and insertion into the plasma membrane – although the interplay and relationship between these mechanisms has not yet been explored. Endocytosis is central to the cellular response to the extracellular environment, controlling receptor distribution on the plasma membrane to modulate signalling, neurotransmitter release and uptake, circulating protein and lipid cargo uptake, and amino acid internalisation for cell proliferation. Uncovering the range of cellular and physiological circumstances in which serotonin regulates endocytosis is of great interest for our understanding of how serotonin regulates mood, and also the fundamental understanding of endocytosis and its regulation throughout the body.

This article has an associated Future Leader to Watch interview with the first author of the paper.

## Abstract (precis)

**Summary:** Serotonin is a neurotransmitter associated usually with our mood, yet recent evidence indicates that serotonin regulates the cell uptake process, endocytosis, diversifying the potential roles of serotonin throughout the body.

## Introduction

Serotonin is widely recognised as a neurotransmitter central to the regulation of mood and perception of the world. However, at least 90% of serotonin is present outside our central nervous system (Gershon and Tack, 2007). Almost every cell type or tissue in the human body expresses at least one of the 13 G-protein-coupled receptor (GPCR) class of serotonin receptors currently identified [Berger et al., 2009; Marin et al., 2020; Masson et al., 2012; The Human Protein Atlas (Uhlén et al., 2015)], and expression of the serotonin transporter, the protein responsible for serotonin uptake into the cell, is near ubiquitous throughout the body [The Human Protein Atlas (Uhlén et al., 2015)]. Outside the central nervous system, serotonin stimulates vasodilation, cell growth, cell migration and mitochondrial biogenesis (Berger et al., 2009; Wyler et al., 2017). Serotonin was first identified as a regulator of endocytosis *in vivo* in 1970 (Ericson et al., 1970). Early studies found serotonin to stimulate phagocytosis in mammalian cells (Sternberg et al., 1987), potential macropinocytic endocytic uptake in murine macrophages (Aviram et al., 1992) and clathrin-dependent endocytosis in Aplysia sea slugs (Bailey et al., 1992; Hu et al., 1993). In conjunction with recent data that have expanded our knowledge of serotonin biology (Dey et al., 2021; Walther et al., 2003), it is evident that serotonin is capable of regulating not only endocytic uptake, but also endocytic sorting through multiple distinct mechanisms. In this Review, we propose that serotonin is a fundamental, and crucially, overlooked regulator of endocytosis and endocytic sorting. The first section of this Review will detail the current body of evidence that demonstrates the mechanisms by which serotonin regulates endocytic uptake and sorting. This will be followed by a focused examination of how each serotonin-dependent mechanism modulates endocytosis, endocytic sorting, and the receptors and cargoes involved. Finally, we will present a unified model of serotonin signalling, serotonylation and receptor-independent regulation, highlighting how they functionally relate to each other in modulating endocytosis.

## A brief primer on endocytosis

### Endocytic pathways of entry into the cell

**Fig. 1.**
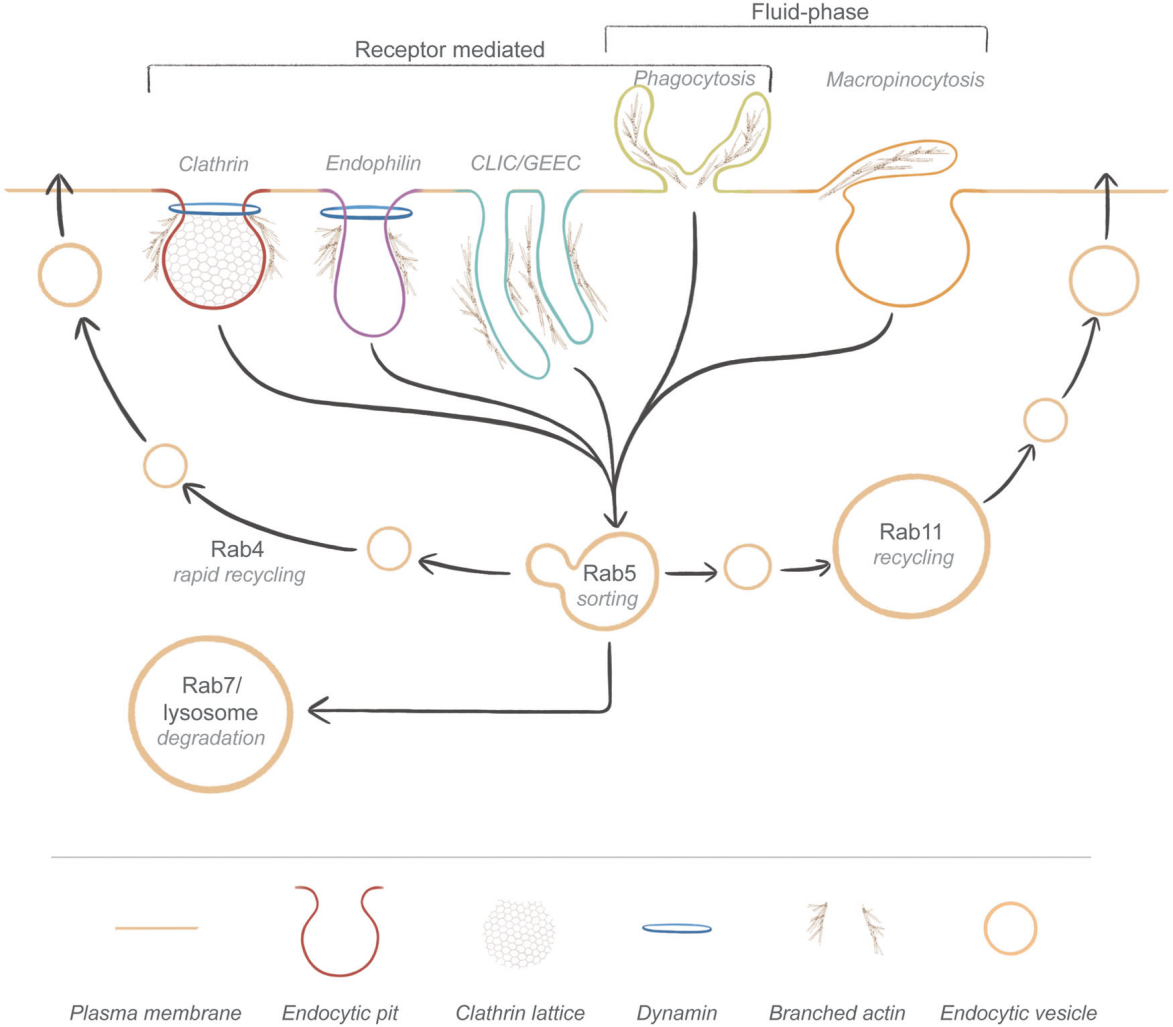
**Endocytic pathways of entry and sorting within the cell.** Clathrin-dependent endocytosis and FEME require dynamin for scission, while actin provides force for membrane deformation to facilitate scission. CLIC/GEEC endocytosis is dynamin independent, with actin leading to endocytic tubule extension and subsequent scission. Macropinocytosis and phagocytosis require extensive actin remodelling, leading to plasma membrane ruffling, forming protrusions that capture cargo for endocytosis. Following endocytosis, cargoes are trafficked to the Rab5^+^ sorting endosome. Cargoes can be rapidly recycled back to the plasma membrane from Rab4^+^ microdomains on the sorting endosome, trafficked to the Rab11^+^ endocytic recycling compartment for ‘slower’ recycling to the plasma membrane or remain in the Rab5^+^ sorting endosome. The Rab5^+^ sorting endosome matures into a Rab7^+^ late endosome, which eventually fuses with the lysosome, degrading remaining cargoes.

Cells rely on endocytosis to modulate cellular signalling responses induced by stimuli from the extracellular environment. Endocytosis removes ligand-bound receptors from the plasma membrane, controls fluid-phase nutrient uptake from the extracellular milieu and regulates receptor density at the plasma membrane, maintaining appropriate concentrations of circulating protein cargoes (Scita and Di Fiore, 2010). Following endocytosis, cargoes and receptors are sorted for degradation or recycling (Jovic et al., 2010). Degradation can reduce receptor numbers on the plasma membrane and/or result in removal of the endocytosed cargo – both limiting cargo uptake and signalling. Conversely, cargo or receptors sorted for recycling are redelivered to the plasma membrane to facilitate continued cargo uptake or receptor signalling or are reused in circulation. Endocytosis and endocytic sorting together facilitate fine-tuned receptor activation and cargo uptake by tightly regulating the levels of receptors at the plasma membrane (Jovic et al., 2010; Naslavsky and Caplan, 2018). To add to this complexity, various mechanisms of endocytic uptake exist (Fig. 1). Each type of endocytic mechanism occurs in a unique plasma membrane environment consisting of a specific membrane lipid composition (Posor et al., 2015; Redpath et al., 2020; Renard and Boucrot, 2021). The precise regulatory mechanisms of endocytosis and endocytic sorting remain to be fully understood (Naslavsky and Caplan, 2018).

Clathrin-dependent endocytosis (Kaksonen and Roux, 2018; Mettlen et al., 2010), fast endophilin-mediated endocytosis (FEME) (Boucrot et al., 2015) and clathrin-independent carrier/glycosylphosphotidylinositol-anchored protein enriched compartments (CLIC/GEEC) endocytosis (Moreno-Layseca et al., 2021; Rossatti et al., 2019) are examples of cargo- or receptor-mediated endocytic mechanisms (Fig. 1). Broadly speaking, these receptor-mediated forms of endocytosis involve ligand-receptor binding that induces endocytic adaptor protein recruitment. The adaptor proteins interact with endocytic coat proteins (clathrin, endophilin or BAR domain-containing proteins for clathrin, fast endophilin and CLIC/GEEC endocytosis, respectively), facilitating endocytic coat formation around the receptor (Boucrot et al., 2015; Chan Wah Hak et al., 2018; Kaksonen and Roux, 2018; Moreno-Layseca et al., 2021; Sathe et al., 2018). This leads to plasma membrane invagination supported by branched, polymerised actin structures (Hinze and Boucrot, 2018). The plasma membrane invagination is extruded, and scission proteins (dynamin for clathrin and fast endophilin, BAR domain-containing proteins for CLIC/GEEC endocytosis) cleave the nascent endosome from the plasma membrane, segregating the receptor and ligand from the membrane (Boucrot et al., 2015; Chan Wah Hak et al., 2018; Kaksonen and Roux, 2018; Moreno-Layseca et al., 2021; Sathe et al., 2018).

Macropinocytosis is another distinct category of endocytosis. It is not driven by ligand-receptor binding; rather, it involves fluid-phase internalisation of extracellular cargo and the surrounding plasma membrane (Fig. 1) (Kerr and Teasdale, 2009). Macropinocytosis occurs either constitutively or is activated by cellular stimuli such as growth factors or amino acid starvation (Canton et al., 2016; Williams and Kay, 2018). Unlike receptor-mediated endocytosis, coat proteins are not reported to be at sites of macropinocytosis. Rather, actin enrichment and reorganisation below the plasma membrane induce the formation membrane ruffles that engulf extracellular cargoes in a wave or pincer-like motion, following which the ruffles collapse back into the plasma membrane, encapsulating and internalising the cargo (Araki et al., 1996, 2007).

Phagocytosis is another distinct type of endocytosis that encompasses aspects of macropinocytosis and receptor-mediated endocytosis (Fig. 1). Phagocytosed stimuli are internalised via large-scale membrane ruffling, leading to cargo engulfment as with macropinocytosis, yet initiation of this membrane ruffling is strictly dependent on receptor engagement by phagocytic cargoes (Mylvaganam et al., 2021). Receptor engagement induces significant actin reorganisation and extension of membrane protrusions called pseudopods, which surround the cargo. The resultant phagosome is closed around the cargo by coordinated action of actin filament enrichment and scission induced by dynamin-2 (Marie-Anaïs et al., 2016), leading to cargo internalisation into the cell (Mylvaganam et al., 2021).

Actin remodelling facilitated by the actin branching complex Arp2/3 is required for each of these modes of endocytosis (reviewed in Hinze and Boucrot, 2018). A suite of actin-regulatory proteins drives clathrin-dependent and clathrin-independent endocytosis. In clathrin-dependent endocytosis, neural Wiskott-Aldrich syndrome protein (N-WASP) activates Arp2/3, leading to actin branching around the forming endosome, which, in conjunction with dynamin and BAR domain-containing proteins, leads to endosome scission from the plasma membrane (Hinze and Boucrot, 2018). Most identified mechanisms of clathrin-independent endocytosis (fast endophilin mediated, CLIC/GEEC, macropinocytosis and phagocytosis) rely on the Rho GTPases RhoA, Rac1 and CDC42 to stimulate endocytosis at the cell leading edge or in membrane ruffles. RhoA activates the actin nucleator Dia1, which provides an initiation point for actin polymerisation required for membrane ruffling (Kurokawa and Matsuda, 2005; Ridley, 2006). Rac1 and CDC42 activate WAVE and N-WASP complex proteins, which in turn activate Arp2/3 (Ridley, 2006). In FEME, the Rho GTPases RhoA, Rac1 and CDC42, as well as Arp2/3 activation, are all required for endosome formation (Boucrot et al., 2015; Chan Wah Hak et al., 2018), although the precise role of actin branching has yet to be elucidated (Hinze and Boucrot, 2018). In CLIC/GEEC endocytosis, CDC42 activates the BAR domain-containing protein IRSp53, which in turn activates Arp2/3 and leads to actin polymerisation at sites of endocytosis to drive dynamin-independent endosome scission (Sathe et al., 2018). In macropinocytosis and phagocytosis, massive membrane remodelling is required to engulf cargoes. Macropinocytosis is highly dependent on calcium influx into the cytoplasm, which activates Rac1 and CDC42 (Canton et al., 2016). Rac1 and CDC42 then activate Arp2/3 via effector proteins to stimulate the actin remodelling required for membrane ruffling (Mylvaganam et al., 2021). In phagocytosis, RhoA stimulates actin fibre nucleation while Rac1 and CDC42 activate Arp2/3, inducing membrane remodelling required for phagocytic engulfment of the cargo (Mao and Finnemann, 2015).

Although actin remodelling is a common feature across all endocytic mechanisms, each type of endocytosis is regulated in a highly specific manner. With respect to the focus of this Review, serotonin signalling and serotonylation activate RhoA, Rac1 and CDC42, and serotonin itself has long been established to stimulate actin polymerisation (Alexander et al., 1987). Serotonin signalling and serotonylation are likely capable of activating multiple mechanisms of clathrin-independent endocytosis, while serotonin insertion into to the plasma membrane can enhance cargo-membrane association.

### Endocytic sorting

The Rab GTPase family of proteins are key mediators of endocytic sorting (Fig. 1). Each Rab protein binds to the membrane of a specific endocytic compartment, recruiting the relevant membrane remodelling proteins, scission factors and molecular motor adaptors required to deliver cargoes to their recycling or degradative fate (Wandinger-Ness and Zerial, 2014). Following endocytosis, endocytic cargoes from all modes of endocytic uptake are delivered to the sorting, or early, endosome decorated with Rab5. Cargoes targeted for different endocytic fates are segregated in microdomains within the Rab5^+^ sorting endosome membrane (Franke et al., 2019). This segregation is maintained in downstream endocytic compartments (Xie et al., 2016), creating functional domains for cargo sorting.

Rab4 and Rab11, both responsible for regulating endocytic recycling, are found to be simultaneously present on Rab5^+^ sorting endosomes, creating specialised microdomains for cargo recycling (De Renzis et al., 2002). Rab4^+^ microdomains facilitate rapid recycling of specific cargoes back to the plasma membrane from the Rab5^+^ sorting endosome (D'Souza et al., 2014; Yudowski et al., 2009), while Rab11^+^ microdomains remove recycling cargoes from Rab5^+^ sorting endosomes for recycling via the Rab11^+^ endocytic recycling compartment (Campa et al., 2018). Cargoes not targeted for Rab4/Rab11-dependent recycling are not removed from the Rab5^+^ sorting endosome; rather, the Rab5^+^ sorting endosome matures into a Rab7^+^ late endosome with the cargo present (Rink et al., 2005). From the Rab7^+^ late endosome, cargo is either recycled via the trans-Golgi compartment via retromer or retriever protein complexes (Burd and Cullen, 2014; McNally et al., 2017), or if it remains, the late endosome fuses with lysosomes and the cargo is degraded (Zhang et al., 2009).

## Serotonin signalling

### A brief overview of serotonin signalling

Serotonin receptors are often studied in the context of neuroscience, yet many serotonin receptors are expressed ubiquitously or highly expressed in tissues outside the brain (Table 1). Fourteen serotonin receptors have been identified in humans, split across seven receptor classes (5-HT_1_ to 5-HT_7_). All serotonin receptors are GPCRs, with the exception of the 5-HT_3­_ class, which heterodimerise to form cation channels that mediate cell depolarisation in response to serotonin (Marin et al., 2020). Serotonin binding to G-protein-coupled serotonin receptors induces a variety of signalling responses (detailed extensively in Sahu et al., 2018). The cellular impacts of serotonin signalling range from cellular proliferation, cell migration, mitochondrial biogenesis and differentiation (Berger et al., 2009).

**Table 1.**
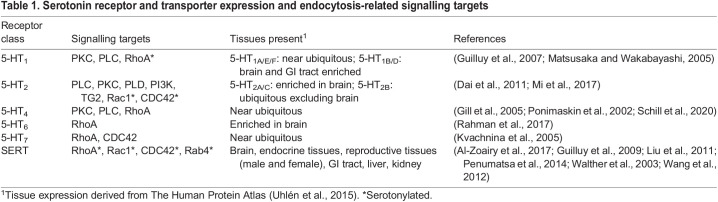
Serotonin receptor and transporter expression and endocytosis-related signalling targets

Serotonin binding to serotonin receptors typically induces receptor endocytosis, terminating signalling (Darmon et al., 2015). In addition to receptor endocytosis, the serotonin transporter (SERT) also acts to modulate serotonin signalling. SERT is a Na^+^/Cl^−^ transporter that internalises serotonin into the cell, depleting extracellular levels and thereby reducing serotonin availability for receptor binding (Baudry et al., 2019). Serotonin signalling through each of 5-HT_1_, 5-HT_2_, 5-HT_4_, 5-HT_6_ and 5-HT_7_ receptors, in some cases in synergy with SERT, has the potential to stimulate endocytosis (Sahu et al., 2018; Table 1), detailed below. As 5-HT_3_ receptors are channels rather than GPCRs and 5-HT_5_ receptors are poorly characterised, they will not be discussed further in this Review.

### Phospholipase C and protein kinase C

**Fig. 2.**
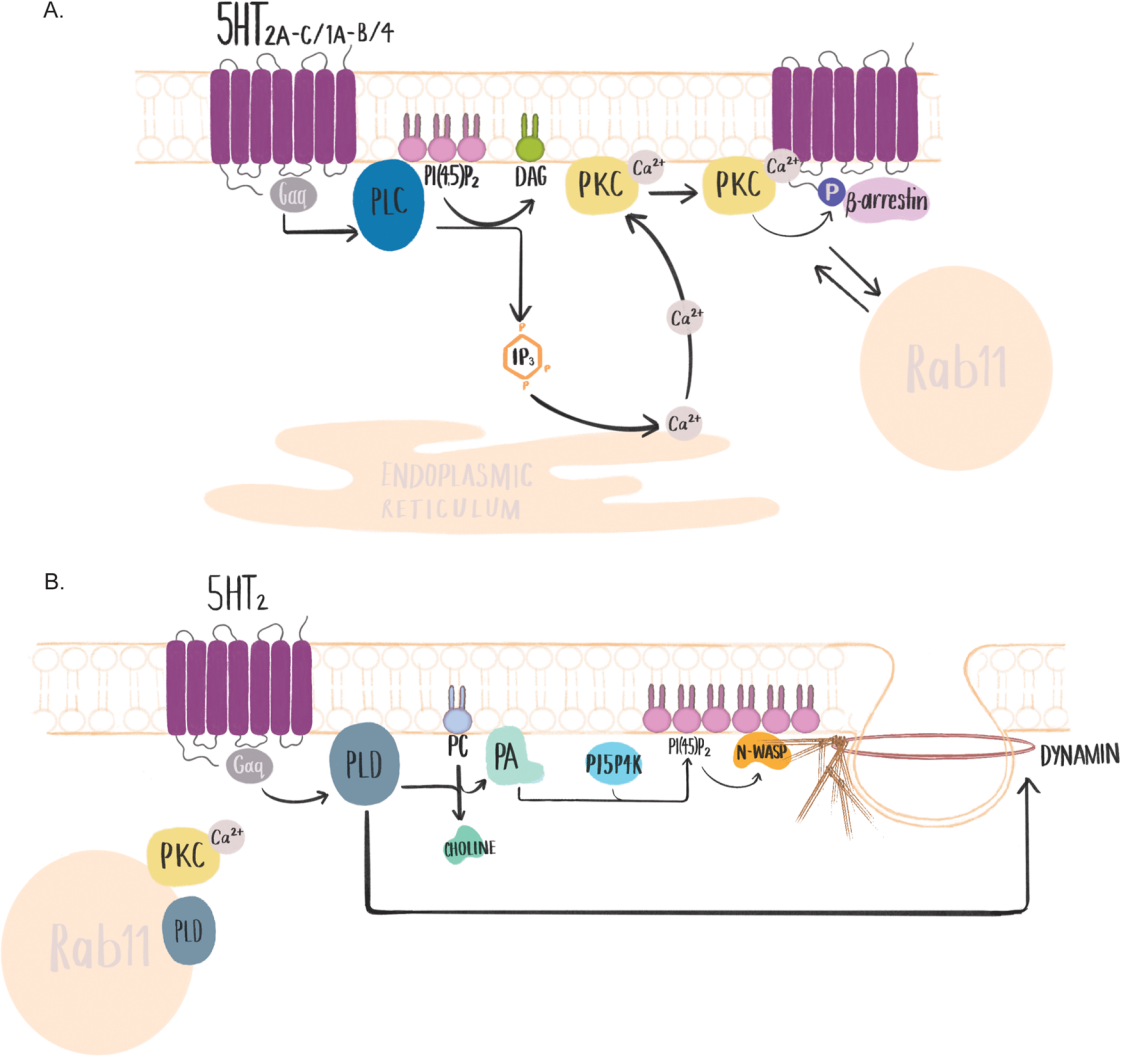
**Serotonin receptor signalling effects on PLC, PKC and PLD.** (A) Serotonin signalling through 5-HT_1A/B_, 5-HT_2A/B/C_ and 5-HT_4_ activates PLC via the G-protein subunit Gαq. PLC catabolises PI(4,5)P2 to DAG and IP3, which induces calcium release from the endoplasmic reticulum. Calcium induces plasma membrane translocation of PKC, where it can phosphorylate GPCR cytoplasmic tails, inducing β-arrestin recruitment. Additionally, PKC can shuttle between the plasma membrane and Rab11^+^ recycling endosomes, where it can modulate endocytic sorting. (B) Serotonin signalling through 5-HT_2_ activates PLD via the G-protein subunit Gαq. PLD catabolises PC to PA and choline. PA activates phosphatidylinositol 5-phosphate 4-kinase, producing PI(4,5)P2. PA also recruits N-WASP to the plasma membrane, where it is activated by PI(4,5)P2, stimulating actin remodelling. PLD also modulates endocytosis and sorting by recruiting dynamin to endosomes and by localising to Rab11^+^ recycling endosomes and being activated in a PKC-dependent manner.

The serotonin receptors 5-HT_1A/B_, 5-HT_2A/B/C_ and 5-HT_4_ (Gill et al., 2005) activate phospholipase C (PLC) via the G-protein subunit Gαq (Dai et al., 2011; Dickenson and Hill, 1998; Fargin et al., 1989; Masson et al., 2012; Raymond et al., 1991) (Fig. 2A). Upon activation, PLC hydrolyses phosphoinositide-(4,5)-phosphate [PI(4,5)P_2_] on the plasma membrane inner leaflet to inositol-1,4,5-triphosphate (IP_3_) and diacylglycerol. IP_3_ induces release of calcium from endoplasmic reticulum stores, while diacylglycerol remains associated with the plasma membrane, activating protein kinase C (PKC) (Lyon and Tesmer, 2013; Newton, 2018). Clathrin-mediated endocytosis is heavily reliant on PI(4,5)P_2_ enrichment in the plasma membrane cytosolic leaflet (Redpath et al., 2020), as such PLC activation decreases activity of this endocytic mode (Carvou et al., 2007; Zoncu et al., 2007). However, many GPCRs, including serotonin receptors, are internalised via clathrin-mediated endocytosis. To prevent inhibition of a GPCR by its own PLC signalling, β-arrestin recruitment to the GPCR leads to enrichment of the PI(4,5)P_2_ synthesis enzyme PIP5K-Iγ, which induces localised production of PI(4,5)P_2_, enabling clathrin-mediated endocytosis of the GPCR to occur (Jung et al., 2021). Serotonin receptor activation of PLC will therefore likely only alter endocytosis of other receptors internalised by clathrin-mediated endocytosis, and, given the localised enrichment of PIP5K to plasma membrane domains and to the clathrin-adaptor AP-2 (Kwiatkowska, 2010), this inhibition is likely to only occur transiently following the initiation of serotonin signalling.

PKC is activated by the 5-HT_2A/B/C_, 5-HT_4_ and 5-HT_7_ receptors (Masson et al., 2012). Following activation, PKC is recruited to the plasma membrane in response to calcium binding to its C2 domain. This prompts PKC to associate with PI(4,5)P_2_ on the membrane inner leaflet. Activated PKC phosphorylates the cytoplasmic tails of multiple transmembrane receptors [epidermal growth factor receptor (EGFR)], channels (for voltage-gated potassium channel Kv1.5) and transporters (SERT, dopamine transporter), inducing their endocytosis (Alvi et al., 2007; Cremona et al., 2011; Du et al., 2021; Ramamoorthy et al., 1998; Santos et al., 2017). GPCRs themselves can be phosphorylated on the C-terminus by PKC or G-protein receptor kinases (Alvi et al., 2007; Jean-Charles et al., 2017). β-arrestin binds phosphorylated tails, leading to clathrin and adaptor protein recruitment to initiate endocytosis (Lin et al., 1997; Shenoy and Lefkowitz, 2011). β-arrestin also acts as a sorting signal adaptor, targeting GPCRs for degradation, rapid recycling or recycling via the endocytic recycling compartment (Alvi et al., 2007; Puthenveedu et al., 2010; Shenoy and Lefkowitz, 2011). Importantly, in the context of PKC phosphorylation and endosomal sorting, activated PKC continuously shuttles to and from the Rab11^+^ endocytic recycling compartment (Becker and Hannun, 2003) and is capable of redirecting degradative cargoes such as EGFR to recycling fates (Bao et al., 2000). Where PKC activation is the result of serotonin stimulation, activated PKC induces the translocation of a range of receptors into the endocytic recycling compartment (EGFR, transferrin, protease-activated receptor, CD59), including those otherwise targeted for degradation (Idkowiak-Baldys et al., 2009).

### Phospholipase D

Phospholipase D (PLD) can be either directly activated by the G-protein subunits of 5-HT_2_ receptors (Masson et al., 2012), or specifically activated in the endocytic recycling compartment by PKC (Becker and Hannun, 2004; Hu and Exton, 2004) (Fig. 2B). Activated PLD is a major source of receptor/signal-generated phosphatidic acid (PA) resulting from phosphatidylcholine (PC) hydrolysis. PA induces changes in membrane curvature (lowering the energy required for membrane fusion/fission) that is required for endocytosis and sorting (Roth, 2008). PLD-generated PA activates phosphatidylinositol 5-phosphate 4-kinase, which generates PI(4,5)P_2_ (Cockcroft, 2009). PA can also recruit the actin nucleator N-WASP to the plasma membrane, which is activated by PI(4,5)P_2_, stimulating actin remodelling required for phagocytosis (Thakur et al., 2019). Increased PI(4,5)P_2_ concentration at the plasma membrane initiates clathrin-mediated endocytosis (Redpath et al., 2020). PLD can also regulate endocytosis by interacting directly with dynamin, recruiting it to EGFR endocytic sites (Lee et al., 2006). PLD also stimulates actin remodelling in macropinocytosis (Mettlen et al., 2006), and PLD activation is required for constitutive and ligand-induced µ-opioid receptor endocytosis (Koch et al., 2006). PLD also facilitates endocytic sorting, enhancing transferrin receptor recycling from Rab11^+^ endocytic recycling compartments, without altering the rate of transferrin endocytosis (Padrón et al., 2006), and expression of catalytically inactive PLD inhibits EGFR degradation (Shen et al., 2001).

### Phospohoinositide-3-kinase

**Fig. 3.**
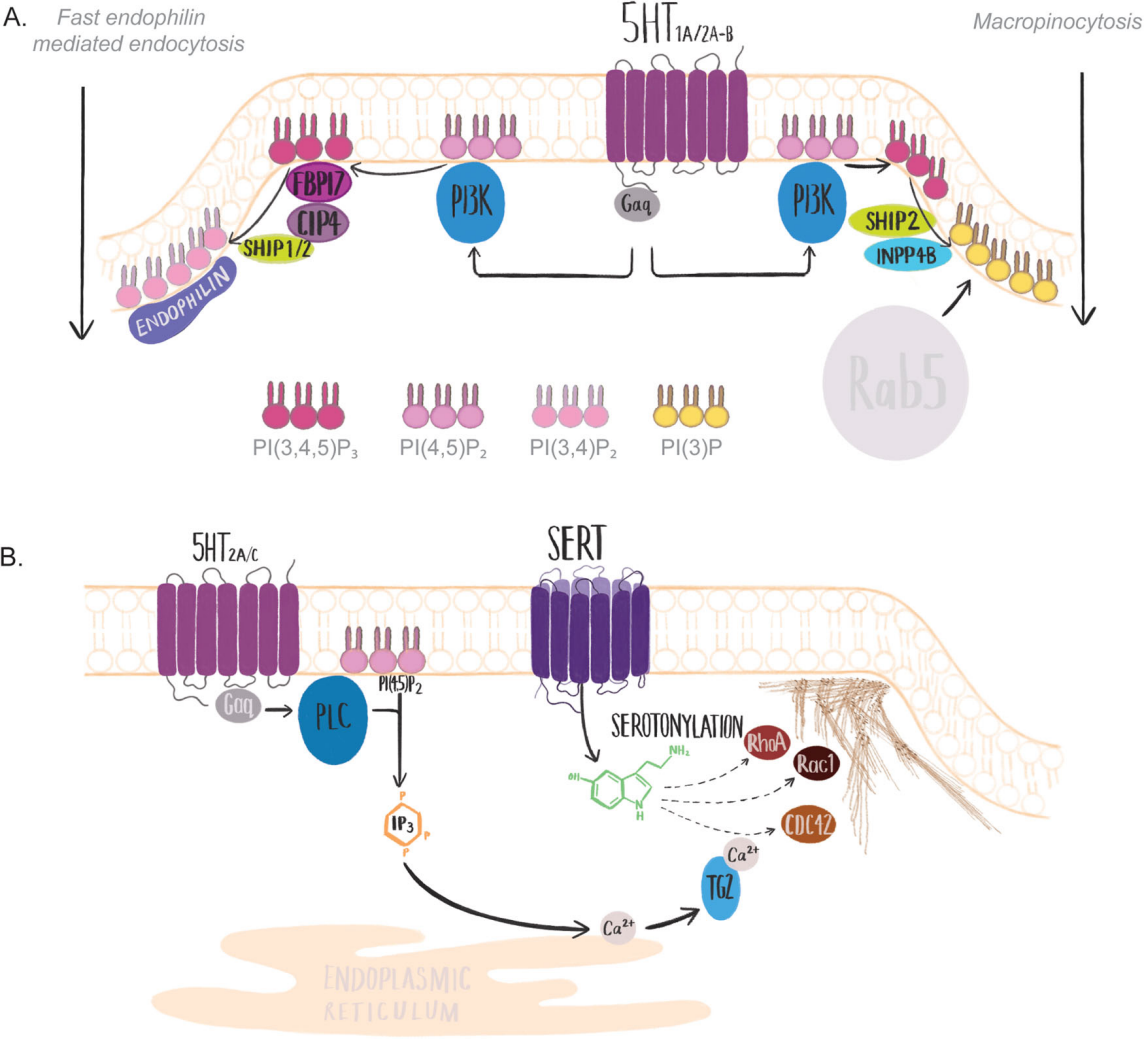
**Serotonin receptor signalling effects on PI3K, RhoA, Rac1 and CDC42.** (A) Serotonin signalling through 5-HT_1A,2A/B_ activates PI3K via Gαq. In FEME (left), PI3K activation phosphorylates PI(4,5)P_2_, generating PI(3,4,5)P_3_, which recruits FBP17, CIP4 and SHIP1/2 to the plasma membrane. SHIP1/2 dephosphorylates PI(3,4,5)P_3_ at the 5′ position, generating PI(3,4)P_2_, recruiting endophilin and initiating endocytosis. In macropinocytosis (right), PI3K activation phosphorylates PI(4,5)P_2_, generating PI(3,4,5)P_3_ and facilitating macropinocytic cup closure. PI(3,4,5)P3 is then dephosphorylated by sequential action of SHIP2 and INPP4B, enriching PI3P on the cytoplasmic face of the macropinosome, allowing cargo entry into Rab5 endosomes. (B) Serotonin signalling through 5-HT_2A/C_ activates PLC via the G-protein subunit Gαq, leading to calcium release from the endoplasmic reticulum. Released calcium activates TG2. Serotonin is internalised into the cell via SERT and is conjugated onto RhoA, Rac1 and potentially CDC42 by TG2. Serotonylated RhoA, Rac1 and CDC42 are then activated and capable of stimulating actin remodelling required for plasma membrane deformation.

Phospohoinositide-3-kinase (PI3K) is activated directly by the G-protein subunits of 5-HT_1A_ and 5-HT_2A/B_ (Masson et al., 2012). While PI3K activation by serotonin receptors leads to Akt activation and signalling (Masson et al., 2012; Sahu et al., 2018), PI3K also regulates multiple modes of clathrin-independent endocytosis by producing phosphoinositide(3,4,5)phosphate [PI(3,4,5)P_3_] from PI(4,5)P_2_ (Araki et al., 2007; Chan Wah Hak et al., 2018; Redpath et al., 2020). Active PI3K is directly required for FEME (Chan Wah Hak et al., 2018), macropinocytosis (Araki et al., 2007) and phagocytosis (Araki et al., 1996). In FEME, PI(3,4,5)P_3_ is required to recruit FBP17 and CIP4, which recruits the phosphatases SHIP1/2 to hydrolyse PI(3,4,5)P_3_ to phosphoinositide(3,4)phosphate [PI(3,4)P_2_]. PI(3,4)P2 is required for endophilin recruitment and for endocytosis to occur (Boucrot et al., 2015; Chan Wah Hak et al., 2018) (Fig. 3A, left). In macropinocytosis, membrane ruffling forms a ‘cup’ in which the cargo is captured. PI(3,4,5)P_3_ is produced from PI(4,5)P_2_ by PI3K on the inner leaflet of forming macropinocytic cups and is further enriched following macropinosome closure (Araki et al., 2007; Yoshida et al., 2009) (Fig. 3A, right). PI(3,4,5)P_3_ is hydrolysed to phosphoinositide(3)phosphate [PI(3)P] by sequential action of SHIP2 and INPP4B (Egami et al., 2014), and is required for Rab5 recruitment to the enclosed macropinosome (Yoshida et al., 2009). In phagocytosis, PI3K generates PI(3,4,5)P_3_ from PI(4,5)P_2_ immediately following receptor binding to the phagocytic cargo (Levin et al., 2015). PI(3,4,5)P_3_ levels increase in the phagocytic cup as it engulfs the cargo, and depletion of PI(3,4,5)P_3_ results in aborted cup formation around the cargo (Levin et al., 2015).

### RhoA

RhoA is activated by G-protein subunits coupled to 5-HT_4_ and 5-HT_7_ (Kvachnina et al., 2005; Ponimaskin et al., 2002; Schill et al., 2020). RhoA can also be activated by 5-HT_6_ signalling (Rahman et al., 2017), and by synergistic activity of 5-HT_1_ receptor signalling and SERT function (Matsusaka and Wakabayashi, 2005) [via serotonylation (Guilluy et al., 2007), discussed below; Fig. 3B]. RhoA activation by each serotonin receptor induces changes in the actin cytoskeleton, leading to changes in cell morphology and inducing cell migration (Kvachnina et al., 2005; Matsusaka and Wakabayashi, 2005; Ponimaskin et al., 2002; Rahman et al., 2017; Schill et al., 2020). RhoA activation induces membrane ruffling (Kurokawa and Matsuda, 2005), and is required for FEME and clathrin-independent endocytosis of the interlukin-2 receptor (Boucrot et al., 2015; Lamaze et al., 2001). 5-HT receptor activation of RhoA establishes a defined leading edge in migrating cells or neurite extension in neuronal cells (Kvachnina et al., 2005; Matsusaka and Wakabayashi, 2005; Ponimaskin et al., 2002; Rahman et al., 2017; Schill et al., 2020), representing a PI(3,4,5)P_3_-enriched environment that facilitates FEME (Boucrot et al., 2015).

### CDC42

CDC42 is directly activated by the Gα subunit coupled to 5-HT_7_ (Kvachnina et al., 2005). CDC42 activation is required for macropinocytosis (Koivusalo et al., 2010), exocytic delivery of endocytic membrane to the plasma membrane in phagocytosis (Mohammadi and Isberg, 2013) and regulation of immunoglobulin-receptor mediated phagocytosis (Caron and Hall, 1998). In CLIC/GEEC endocytosis, transient activation of CDC42 results in recruitment of the CLIC/GEEC regulator GRAF1 and induction of actin polymerisation, followed by CDC42 inactivation and dissociation from GRAF1 endocytic tubules and CLIC maturation (Francis et al., 2015). In FEME, CDC42 activation is required for the recruitment of the FEME effectors SHIP2, lamellipodin and endophilin. Rounds of CDC42 activation and deactivation are essential for constant assembly and disassembly of membrane patches primed for FEME (Boucrot et al., 2015; Chan Wah Hak et al., 2018).

### The effect of serotonin signalling on cargo endocytosis and sorting

Several receptors and cargoes have been identified to be endocytosed in response to the effects of serotonin signalling. Endocytosis of 5-HT_1A_, 5-HT_2A/B/C_ and 5-HT_4_ receptors is stimulated by serotonin and subsequent activation of PKC/IP3 generation (Bhattacharyya. et al., 2002; Della Rocca et al., 1999; Mnie-Filali et al., 2010; Porter et al., 2001; Riad et al., 2001; Schlag et al., 2004). Beyond serotonin receptor endocytosis, serotonin signalling stimulates the endocytosis and delivery of a large array of receptors to the Rab11^+^ endocytic recycling compartment (Idkowiak-Baldys et al., 2009). When HEK293 cells overexpressing 5-HT_2A_ are treated with serotonin, it stimulates the endocytosis of EGFR and the protease-activated receptor even in the absence of ligand. Serotonin activation of PKC and PLD induced redistribution of EGFR to the endocytic recycling compartment and inhibited receptor degradation, causing a change in the endocytic fate of the receptor (Idkowiak-Baldys et al., 2009). Serotonin also enhanced the levels of endocytosis and targeting of transferrin and CD59 to the endocytic recycling compartment (Idkowiak-Baldys et al., 2009). Both transferrin and CD59 cargoes are typically targeted for Rab11-dependent recycling, but internalised by clathrin-dependent endocytosis and CLIC/GEEC endocytosis, respectively (Cai et al., 2011; Naslavsky et al., 2004). Clearly, serotonin receptor activation of PKC and PLD not only stimulates the endocytosis of receptor cargoes through diverse endocytic mechanisms but also redistributes cargoes to the endocytic recycling compartment and inhibits cargo degradation. Direct demonstration of serotonin signalling-induced activation RhoA/CDC42 mediated actin remodelling and subsequent changes in cargo endocytosis will firmly establish serotonin signalling as a modulator of endocytosis.

## Serotonylation

### Endocytic regulators targeted by serotonylation

In addition to inducing receptor signalling, serotonin directly alters the activity of intracellular proteins via serotonylation. Serotonylation is the process by which cytoplasmic serotonin is conjugated to a protein, leading to activation of GTPases important for actin remodelling (RhoA, Rac1 and CDC42; Fig. 3B, Table 2) and endocytic sorting facilitating rapid recycling (Rab4) (Walther et al., 2003).

**Table 2.**
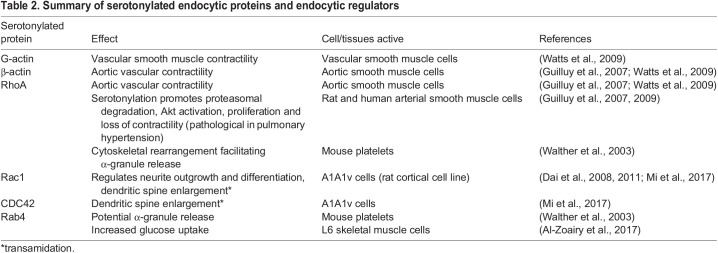
Summary of serotonylated endocytic proteins and endocytic regulators

Serotonylation is the conjugation of a serotonin molecule onto specific glutamate residues at a conserved consensus sequence in RhoA, Rac1, CDC42, Rab4 by the widely expressed enzyme transglutaminase 2 (TG2) (Walther et al., 2003). TG2 is a calcium-activated enzyme (Hand et al., 1985), with increases in cytoplasmic calcium caused by PLC-induced generation of IP_3_, inducing activation (Nurminskaya and Belkin, 2012). Serotonylation predominantly utilises serotonin entering the cell via SERT (Al-Zoairy et al., 2017; Guilluy et al., 2009; Liu et al., 2011; Penumatsa et al., 2014; Walther et al., 2003; Wang et al., 2012; Watts et al., 2009). Serotonin receptor activation of PLC activates TG2 and serotonylation (Dai et al., 2008, 2011), indicating that serotonin signalling and uptake act in concert to induce serotonylation.

In the following sections, we detail the effects serotonylation has on RhoA, Rac1, CDC42, Rab4 and actin. Although serotonylation has not been directly linked to endocytosis, current evidence suggests that a link is likely. We outline the potential consequences serotonylation of GTPases has on endocytosis and sorting, and the cargoes identified in which serotonylation modulates their endocytic uptake or sorting.

### RhoA

Serotonylation constitutively activates RhoA (Walther et al., 2003), which stimulates α-granule exocytosis in platelets (Walther et al., 2003), pulmonary artery (smooth muscle) contraction (Guilluy et al., 2009), and pulmonary artery remodelling and RhoA translocation to the plasma membrane in lung tissue (Wang et al., 2012) (Table 2). Serotonylation also targets RhoA for proteasomal degradation, which finally results in inhibition of smooth muscle contraction (Guilluy et al., 2007). RhoA membrane translocation and activation is observed in membrane ruffles at the cell leading edge (Kurokawa and Matsuda, 2005); therefore, RhoA serotonylation has the potential to stimulate leading edge endocytic mechanisms [endophilin mediated (Boucrot et al., 2015), CLIC/GEEC (Sathe et al., 2018)] or membrane ruffling [macropinocytosis (Araki et al., 2007)].

### Rac1

Rac1-induced membrane remodelling and ruffling is required for clathrin-independent endocytosis and macropinocytosis. 5-HT_2A/C_ receptor signalling activates Rac1 via serotonylation (Dai et al., 2011; Mi et al., 2017). 5-HT_2_ receptor stimulation activates PLC, which prompts calcium release, transglutaminase-2 activation and Rac1 serotonylation, activating Rac1 (Dai et al., 2011). Serotonylation transiently activates Rac1, and subsequent deactivation does not rely on degradation, unlike serotonylated RhoA (Dai et al., 2008). Membrane remodelling and ruffling in response to Rac1 activation are driven by Arp2/3 actin branching, which itself is activated via the WAVE complex (Chen et al., 2017; Renard and Boucrot, 2021; Römer et al., 2010). Transient Rac1 activation is required for macropinocytosis, with activation stimulating membrane ruffling and deactivation, leading to ruffle retraction, internalising engulfed cargo into the cell (Fujii et al., 2013). Rac1 activation is also required for FEME (Boucrot et al., 2015), clathrin-independent endocytosis of the interlukin-2 receptor (Grassart et al., 2008) and immunoglobulin receptor-mediated phagocytosis (Caron and Hall, 1998).

### CDC42

CDC42 has not been directly shown to be serotonylated, but shares the serotonylation consensus sequence of RhoA, Rac1 and Rab4 (Walther et al., 2003), and is activated by serotonin receptor-stimulated transamidation (Mi et al., 2017). Serotonin receptor-stimulated CDC42 transamidation stimulates dendritic spine formation in neurons (Mi et al., 2017), consistent with actin remodelling typically stimulated by CDC42 and required for macropinocytosis, FEME, CLIC/GEEC endocytosis and phagocytosis (Renard and Boucrot, 2021).

### Actin

Actin was first described as a serotonin-binding protein in 1984 (Small and Wurtman, 1984), and multiple studies have subsequently identified actin as a serotonylation target (Lin et al., 2014; Watts et al., 2009). Inhibition of transglutaminase reduces the contractility of rat thoracic aorta, indicating that actin serotonylation may increase actin contractile properties (Watts et al., 2009). Serotonin increases actin polymerisation in endothelial cells and is incorporated into actin fibres (Alexander et al., 1987), indicating that such polymerisation is serotonylation induced. Actin polymerisation and reorganisation is required for all forms of endocytosis to aid membrane remodelling and provide mechanical force for scission of the endosome from the plasma membrane (Boucrot et al., 2015; Hinze and Boucrot, 2018; Renard and Boucrot, 2021; Sathe et al., 2018). The increase in contractile properties of actin induced by serotonin, in conjunction with serotonylation activating actin-remodelling factors, indicates that actin serotonylation may play a crucial regulatory role in endocytosis.

### Sorting regulators targeted by serotonylation

Rab4 is serotonylated following serotonin treatment in myoblast cell lines (Al-Zoairy et al., 2017). Serotonin stimulates glucose uptake and glucose transporter (GLUT4) translocation to the plasma membrane (Al-Zoairy et al., 2017). Rab4 is well established to stimulate GLUT4 translocation to the plasma membrane in response to insulin (Kaddai et al., 2008; Mari et al., 2006). Assuming that serotonylation activates Rab4 as with other GTPases, Rab4 serotonylation may stimulate GLUT4 plasma membrane translocation by fulfilling its role as a mediator of rapid recycling from sorting endosomes (D'Souza et al., 2014), delivering GLUT4 to the plasma membrane.

### Can serotonylation modulate endocytic uptake and sorting?

Although there is currently little direct evidence that serotonylation modulates endocytic uptake or sorting, the currently identified GTPase serotonylation targets strongly suggest that this is likely (Table 2). RhoA, Rac1 and CDC42 are well-established mediators of actin remodelling for multiple modes of clathrin-independent endocytosis (Hinze and Boucrot, 2018; Renard and Boucrot, 2021); therefore, their serotonylation could potentially stimulate clathrin-independent endocytosis, and actin serotonylation may further facilitate this actin remodelling. Rab4 is an established regulator of rapid cargo recycling (D'Souza et al., 2014), indicating that serotonylation may be able to facilitate endocytic sorting. SERT is widely expressed [Table 1; The Human Protein Atlas (Uhlén et al., 2015)], while TG2 is broadly expressed throughout the body, indicating that serotonylation could occur widely throughout the body [The Human Protein Atlas (Uhlén et al., 2015)]. GTPase serotonylation has currently only been investigated in a limited range of cells and issues (Table 2); therefore, the potential effects of serotonylation on cargo endocytosis and sorting remain to be discovered.

## Receptor- and transporter-independent effects of serotonin

Serotonin insertion into the plasma membrane is a recently described mechanism of serotonin action. When serotonin inserts into the plasma membrane, it enhances the membrane binding of diverse cargoes and increases their subsequent internalisation (Dey et al., 2021).

Serotonin is an amphipathic molecule that non-specifically associates with membranes at physiological concentrations *in vitro*. Serotonin inserts into the membrane bilayer below the phospholipid headgroup without disrupting bilayer integrity (Josey et al., 2020), leading to changes in overall membrane order, as well as decreasing phospholipid chain order and physical length (Dey et al., 2021). Serotonin insertion into ordered domains further increases membrane order, and insertion into disordered membrane domains further decreases membrane order (Engberg et al., 2020). Serotonin nucleates formation of disordered domains, resulting in decreased membrane stiffness and reduced membrane surface tension (Dey et al., 2021). Reduced membrane tension facilitates CLIC/GEEC endocytosis in a range of adherent cell lines (Thottacherry et al., 2018), while increased membrane tension inhibits multiple forms of endocytosis in neurons (Wu et al., 2017). These serotonin-induced membrane changes are capable of modulating endocytosis and support the concept that membrane order changes play a role in serotonin exerting its functional effects.

Physiological concentrations of serotonin added to serotonergic neurons increases binding of islet amyloid precursor protein to the plasma membrane and increases transferrin endocytosis. These experiments were conducted in the presence of serotonin receptor and transporter inhibitors (Dey et al., 2021), strongly suggesting that changes in serotonin levels on/in the plasma membrane enhance endocytic uptake. Corroborating these findings are our recent experiments showing that plasma membrane binding and endocytosis of the circulating lipoprotein, lipoprotein(a) [Lp(a)], is potentially enhanced independent of serotonin receptors and transporter (Redpath et al., 2021 preprint). Treatment with the antidepressants (SERT inhibitors) imipramine and citalopram, or serotonin itself, significantly increased Lp(a) binding to the plasma membrane and subsequent endocytosis into HepG2 liver cells in a macropinocytosis-dependent manner (Redpath et al., 2021 preprint). HepG2 cells express only trace levels of 5-HT_1A_ and 5-HT_1D_ receptors [The Human Protein Atlas (Thul et al., 2017)], indicating that serotonin receptor and transporter-independent effects are enhancing Lp(a) plasma membrane binding and uptake.

Serotonin predominantly inserts into the exposed outer membrane leaflet when added to membrane bilayers *in vitro*, increasing the size or level of disorder in lipid domains (Josey et al., 2020). Alterations to membrane order on one side of a lipid bilayer have been shown to translate into order changes on the other (Fujimoto and Parmryd, 2017); therefore, serotonin insertion into the extracellular face of the plasma membrane may translate into changes in membrane order on the cytoplasmic face. To test the functional consequences further, it would be useful to examine the preservation of the changes in membrane order (i.e. lipid disorder) in the endosomes that subsequently form. Rab5 has been reported to bind to disordered lipid bilayers *in vitro* (Kulakowski et al., 2018). Although it is not yet clear if the state of plasma membrane order can directly translate from one leaflet of the membrane to the other (Fujimoto and Parmryd, 2017), it is tempting to speculate that serotonin-induced lipid disorder could modulate recruitment of endocytic sorting regulators. In our recent study, we found that imipramine and citalopram treatment enhanced Lp(a) delivery to Rab11^+^ recycling endosomes (Redpath et al., 2021 preprint), indicating that serotonin-induced membrane order changes may translate into functional differences of endosomal cargo sorting, although this remains to be directly tested with serotonin treatment.

## Temporal and stoichiometric considerations

In evaluating the likelihood of serotonin to induce endocytosis through serotonylation and membrane insertion in a physiological relevant manner, temporal and stoichiometric considerations must be examined. *In vivo*, plasma (excluding platelets) serotonin levels are ∼1-3 nM (Brand and Anderson, 2011). The half-maximal effective concentration (EC_50_) of 5-HT_2A_ is 8.09 nM and that of 5-HT_2C_ is 9.87 nM (Toro-Sazo et al., 2019), while the Michaelis constant (Km) of the serotonin affinity of SERT is 463 nM (Ramamoorthy et al., 1993). Serotonin signalling is therefore likely to predominate, albeit to a very limited degree, in conditions with basal serotonin levels, with little serotonin available for entering the cell or binding the plasma membrane.

High concentrations of serotonin are stored in cell types such as serotonergic neurons and platelets. The concentration of serotonin is 270 mM in leech synaptic vesicles [4700 serotonin molecules per vesicle (Bruns and Jahn, 1995)]. In RN46A serotonergic cell line synaptic vesicles, the serotonin concentration is 400 mM (Balaji et al., 2005), and 63 mM in platelet dense granules (Herr et al., 2017), equating to ∼7000-58,000 serotonin molecules per vesicle/granule given the sizes of synaptic vesicles and dense granules (diameter of 40 nm compared to 150 nm, respectively) (Flaumenhaft and Sharda, 2018; Schikorski, 2014). Serotonin vesicle/granule exocytosis would therefore induce high, localised increases in serotonin levels in neuronal and peripheral tissues.

Following vesicle release, all three modes of serotonin action would be likely to work in concert. Serotonin receptors would be rapidly activated, resulting in intracellular calcium release peaking within 1 min of receptor activation, and RhoA activation peaking within 2.5 min (Tany et al., 2021 preprint). Calcium release would allow activation of TG2 (Dai et al., 2011), indicating that serotonylation could occur within 1 min of receptor activation. The transport rate of SERT ranges from 1 pM/min/mg of protein in platelets (Singh et al., 2013) to 162 pM/min/mg of protein in neuronal synaptosomes (Perez et al., 2007), equating to ∼2300-368,000 molecules of serotonin/cell/min. A single site of endocytosis contains ∼5700 actin molecules and 200 Arp2/3 complexes (Akamatsu et al., 2020), indicating that, with vesicular serotonin release and the transport rate into the cell, serotonylation is likely to activate a reasonable proportion of Arp2/3 (via Rac1/CDC42) and modify actin at endocytic sites. Finally, serotonin has a high affinity for membranes (Josey et al., 2020), meaning that at high local concentrations it would rapidly insert into the plasma membrane (Dey et al., 2021). Serotonin exposure in both Dey et al. (2021) and our study (Redpath et al., 2021 preprint) was over a timescale of minutes to hours, a longer timeframe of serotonin exposure than would likely be caused by serotonin vesicle release. These studies show that serotonin is clearly capable of enhancing cargo binding and endocytosis, but it remains to be determined how acute versus chronic serotonin treatment modulates endocytosis.

## A unifying hypothesis

Serotonylation, serotonin-induced membrane changes and serotonin signalling all have potential effects on endocytic uptake and sorting. Serotonin receptors, and SERT, are widely expressed throughout the body (Table 1), while serotonin non-specifically binds cellular plasma membranes. It is therefore unlikely that each mechanism of serotonin action acts in isolation, rather serotonin signalling, serotonylation and serotonin membrane binding likely act on a cell simultaneously. While serotonin signalling and serotonylation activate a range of GTPases required for clathrin-independent endocytic mechanisms (Tables 1 and 2), some effects of serotonin may be capable of enhancing clathrin-mediated endocytosis (Cockcroft, 2009), illustrated by the enhanced uptake of the clathrin-dependent cargo transferrin by serotonin membrane binding (Dey et al., 2021).

We hypothesise that these three serotonin mechanisms act synchronously to balance cargo uptake via multiple endocytic pathways to ultimately facilitate downstream effects of serotonin signalling. As an example, serotonin signalling through the 5-HT_7_ receptor enhances T-cell activation (León-Ponte et al., 2007). Upon T-cell activation, rapid, CDC42-dependent endocytosis and recycling of the T-cell receptor occurs (Rossatti et al., 2019), facilitating T-cell receptor signalling and T-cell activation (Compeer et al., 2018). T-cell activation induces proliferation (Hwang et al., 2020), and PI3K and Rac1 activated macropinocytosis delivers the amino acids required for this proliferation (Charpentier et al., 2020). Serotonin signalling and serotonylation could upregulate the endocytic pathways required for T-cell activation and the amino acid acquisition required for subsequent proliferation. However, iron (from transferrin) is required for co-signalling needed for complete T-cell activation, without which proliferation does not occur (Kuvibidila et al., 2003). Here, the membrane-binding effects of serotonin may come in to play. By enhancing the cell surface binding of transferrin (Dey et al., 2021), serotonin could increase iron delivery into T cells by either enhancing transferrin receptor binding, or by increasing transferrin macropinocytosis. This example illustrates how each mechanism of serotonin action could converge to drive the cellular outcomes of serotonin signalling.

## Conclusions

Serotonin is clearly capable of enhancing endocytosis and endocytic sorting through serotonin receptor signalling, serotonylation and membrane binding. The precise endocytic uptake mechanism(s) enhanced by serotonin and the endocytic sorting pathways it modulates have yet to be molecularly defined. Given the widespread expression of serotonin receptors, and the transporter- and membrane-binding effects, the range of cargoes modulated by the effects of serotonin on endocytic and sorting is likely to be large. We can gain a much deeper understanding of the mechanics of endocytosis through understanding serotonin and serotonin signalling in the context of endocytosis.

With the widespread presence of serotonin and serotonin receptors, it is being increasingly recognised that serotonin has a wide range of effects throughout the body (Berger et al., 2009; Gershon and Tack, 2007; Wyler et al., 2017), and, with particular reference to the role of serotonin on endocytic processes, these effects could have extremely wide and varied impact on normal human physiology. What this means in terms of the biology of functions in which serotonin is thought to play a central role – our perception of the world, our mental health, immunity and gastrointestinal function – has yet to be elucidated. Understanding the impact of serotonin on endocytosis and how this plays into these physiological functions and associated conditions, such as depression, anxiety and inflammatory bowel syndrome, will be an important step in improving human health.
